# A Zeaxanthin-Producing Bacterium Isolated from the Algal Phycosphere Protects Coral Endosymbionts from Environmental Stress

**DOI:** 10.1128/mBio.01019-19

**Published:** 2020-01-21

**Authors:** Keisuke Motone, Toshiyuki Takagi, Shunsuke Aburaya, Natsuko Miura, Wataru Aoki, Mitsuyoshi Ueda

**Affiliations:** a Graduate School of Agriculture, Kyoto University, Kyoto, Japan; b Japan Society for the Promotion of Science, Kyoto, Japan; c Atmosphere and Ocean Research Institute, The University of Tokyo, Kashiwa, Japan; d Graduate School of Life and Environmental Sciences, Osaka Prefecture University, Sakai, Japan; e Core Research for Evolutional Science and Technology (CREST), Japan Science and Technology Agency (JST), Tokyo, Japan; Max Planck Institute for Marine Microbiology

**Keywords:** *Symbiodiniaceae*, antioxidant, coral, microbiome, stress tolerance, zeaxanthin

## Abstract

Reef-building corals form a complex consortium with photosynthetic algae in the family *Symbiodiniaceae* and bacteria, collectively termed the coral holobiont. These bacteria are hypothesized to be involved in the stress resistance of the coral holobiont, but their functional roles remain largely elusive. Here, we show that cultured *Symbiodiniaceae* algae isolated from the reef-building coral Galaxea fascicularis are associated with novel bacteria affiliated with the family *Flavobacteriaceae*. Antibiotic treatment eliminated the bacteria from cultured *Symbiodiniaceae*, resulting in a decreased maximum quantum yield of PSII (variable fluorescence divided by maximum fluorescence [F_v_/F_m_]) and an increased production of reactive oxygen species (ROS) under thermal and light stresses. We then isolated this bacterial strain, named GF1. GF1 inoculation in the antibiotic-treated *Symbiodiniaceae* cultures restored the F_v_/F_m_ and reduced the ROS production. Furthermore, we found that GF1 produces the carotenoid zeaxanthin, which possesses potent antioxidant activity. Zeaxanthin supplementation to cultured *Symbiodiniaceae* ameliorated the F_v_/F_m_ and ROS production, suggesting that GF1 mitigates thermal and light stresses in cultured *Symbiodiniaceae* via zeaxanthin production. These findings could advance our understanding of the roles of bacteria in *Symbiodiniaceae* and the coral holobiont, thereby contributing to the development of novel approaches toward coral protection through the use of symbiotic bacteria and their metabolites.

## Abstract (precis)

Occupying less than 1% of the seas, coral reefs are estimated to harbor ∼25% of all marine species. However, the destruction of coral reefs has intensified in the face of global climate changes, such as rising seawater temperatures, which induce the overproduction of reactive oxygen species harmful to corals. Although reef-building corals form complex consortia with bacteria and photosynthetic endosymbiotic algae of the family *Symbiodiniaceae*, the functional roles of coral-associated bacteria remain largely elusive. By manipulating the *Symbiodiniaceae* bacterial community, we demonstrated that a bacterium that produces an antioxidant carotenoid could mitigate thermal and light stresses in cultured *Symbiodiniaceae* isolated from a reef-building coral. Therefore, this study illuminates the unexplored roles of coral-associated bacteria under stressful conditions.

## INTRODUCTION

Coral reefs are among the most diverse and productive ecosystems on the globe, providing energy and habitats for nearly 25% of all marine species (1, 2). Recently, the destruction of coral reefs has been exacerbated worldwide, and coral protection is now an immediate concern in the face of global climate change (3–5). Reef-building corals are characterized by the formation of a holobiont associated with endosymbiotic dinoflagellates in the family *Symbiodiniaceae* and other microorganisms, such as bacteria and fungi (6). In the coral holobiont, *Symbiodiniaceae* translocate photosynthetic products to the coral host, which, in turn, supplies carbon dioxide and inorganic nutrients to algal endosymbionts (7). However, this symbiotic relationship is fragile and believed to collapse (i.e., coral bleaching) through the overproduction of reactive oxygen species (ROS) in the endosymbiotic algae because of thermal and light stresses (8). Prolonged bleaching ultimately leads to death unless the corals can reconstitute their association with *Symbiodiniaceae*, especially those with a high stress tolerance (9). Therefore, understanding and enhancing the stress resistance of *Symbiodiniaceae* is important for sustaining coral holobiont health under stressful conditions, as demonstrated by several studies (10, 11).

Recently, the beneficial roles of bacteria in the coral holobiont have been investigated (6). These functions include defense against pathogens (12, 13) and the cycling of nutrients such as nitrogen (14) and sulfur (15). Moreover, coral-associated bacteria have been proposed to be involved in coral resistance to bleaching (16, 17), which has a devastating impact on reefs worldwide (4). Although some bacterial species are intimately associated with corals and cultured *Symbiodiniaceae* (18–20), suggesting their importance in the coral holobiont, functional and molecular assessments of such bacteria are still in their infancy, partly because corals are limited in their amenability to microbiome manipulation (6).

In this study, we used cultured *Symbiodiniaceae* isolated from the reef-building coral Galaxea fascicularis to disentangle the complex interactions between *Symbiodiniaceae* and bacteria. In manipulating this bacterial community, we identified a bacterium that improves the thermal and light tolerances of cultured *Symbiodiniaceae*. Furthermore, we investigated the mechanisms underlying the observed increased stress resistance of cultured *Symbiodiniaceae*. This study demonstrates the importance of alga-bacterium interactions, paving the way toward a better understanding of the bacterial contribution to coral holobiont physiology under stressful conditions.

## RESULTS

### Bacterial community affects stress tolerance of cultured *Symbiodiniaceae*.

**FIG 1:**
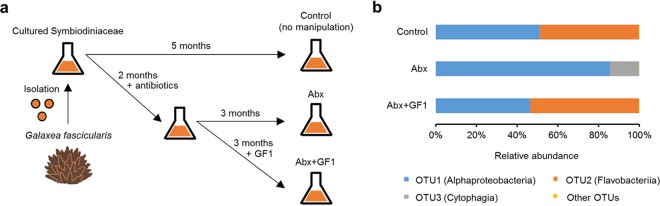
Bacterial community analysis of cultured *Symbiodiniaceae*. (a) *Symbiodiniaceae* were isolated from *G. fascicularis* and cultured in the absence (control) or presence of antibiotics. The antibiotic-treated *Symbiodiniaceae* were subsequently incubated in the absence of antibiotics without (Abx) or with (Abx+GF1) inoculation of the bacterial strain GF1 (see Fig. 3a and b). (b) Bacterial community composition based on 16S rRNA amplicon sequencing. Data were provided as the means of relative abundances from three biological replicates. OTU, operational taxonomic unit.

To investigate the relationship between the bacterial community and the stress tolerance of *Symbiodiniaceae*, we isolated and cultured *Symbiodiniaceae* from *G. fascicularis* (Fig. 1a). The bacterial community of the cultured *Symbiodiniaceae* (i.e., the control) was dominated by bacteria affiliated with the classes *Alphaproteobacteria* and *Flavobacteriia* (Fig. 1b). To perturb this bacterial community, we administered an antibiotic mixture (50 μg/ml of kanamycin, 100 μg/ml of ampicillin, and 50 μg/ml of streptomycin) (21) to the algal culture (Abx), resulting in the elimination of operational taxonomic unit 2 (OTU2) (*Flavobacteriia*) sequences and an increase in the relative abundance of bacteria affiliated with the class *Cytophagia* (Fig. 1b).

**FIG 2:**
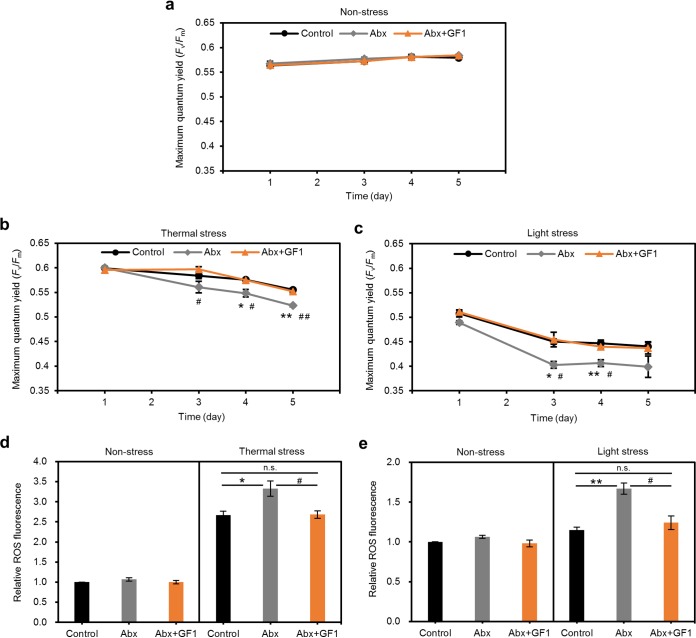
Effects of bacterial community on thermal and light tolerance of cultured *Symbiodiniaceae*. Maximum quantum yield of PSII (F_v_/F_m_) in cultured *Symbiodiniaceae* under nonstress (24°C, 50 μmol photons m^−2^ s^−1^) (a), thermal stress (31.5°C, 50 μmol photons m^−2^ s^−1^) (b), and light stress (24°C, 200 μmol photons m^−2^ s^−1^) (c) conditions. Relative ROS production in cultured *Symbiodiniaceae* after 5 days of exposure to thermal (31.5°C, 50 μmol photons m^−2^ s^−1^) (d) or light (24°C, 200 μmol photons m^−2^ s^−1^) (e) stress. Data ae provided as the relative fluorescence to that of control under nonstress conditions (24°C, 50 μmol photons m^−2^ s^−1^). Error bars indicate standard errors of the means (SEMs) from three biological replicates, and significant differences were determined by Tukey’s *post hoc* tests. *, *P* < 0.05; **, *P* < 0.01 (between control and Abx), #, *P* < 0.05, ##, *P* < 0.01 (between Abx and Abx+GF1). Control, cultured *Symbiodiniaceae* without manipulation of bacterial community; Abx, cultured *Symbiodiniaceae* treated with antibiotics; Abx+GF1, cultured *Symbiodiniaceae* treated with antibiotics followed by inoculation of the bacterial strain GF1.

To examine the effects of the altered bacterial community on the stress tolerance of the cultured *Symbiodiniaceae*, we exposed algal cultures to thermal stress (at a temperature of 31.5°C and a normal light intensity of 50 μmol photons m^−2^ s^−1^) and light stress (at a temperature of 24°C and a high light intensity of 200 μmol photons m^−2^ s^−1^). Consequently, the Abx group showed a significantly lower maximum quantum yield of PSII (variable fluorescence divided by maximum fluorescence [F_v_/F_m_]) and increased ROS production under thermal and light stresses, whereas no significant differences in the F_v_/F_m_ and ROS production were observed under nonstressful conditions between the control and Abx groups (Fig. 2a to e).

**FIG 3:**
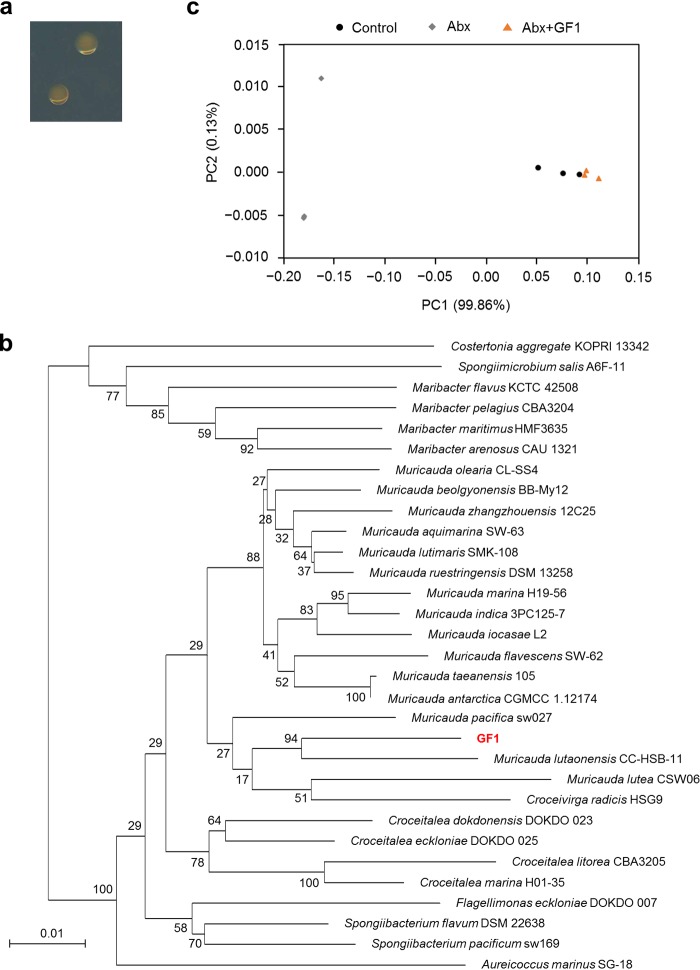
Isolation and inoculation of GF1. (a) GF1 colonies grown on a marine agar plate. (b) Neighbor-joining tree based on 16S rRNA gene sequences showing the phylogenetic relationships of GF1 and related taxa in the family *Flavobacteriaceae*. Numbers on branches represent bootstrap values (1,000 replications). Bar, 0.01 substitutions per nucleotide position. (c) Principal-coordinate analysis of weighted UniFrac distances of bacterial community composition. Control, cultured *Symbiodiniaceae* without manipulation of bacterial community; Abx, cultured *Symbiodiniaceae* treated with antibiotics; Abx+GF1, cultured *Symbiodiniaceae* treated with antibiotics followed by inoculation of the bacterial strain GF1.

To confirm that these changes in the stress tolerance of *Symbiodiniaceae* were induced in a manner dependent on the abundance of OTU2, we isolated a bacterium (named GF1) with a 16S rRNA gene sequence identical to the representative sequence of OTU2 from the *Symbiodiniaceae* culture (Fig. 3a). The 16S rRNA sequence of GF1 showed 95.7% similarity to that of the most closely related strain, Muricauda lutaonensis CC-HSB-11 (22, 23), which belongs to the family *Flavobacteriaceae* (Fig. 3b). We inoculated antibiotic-treated *Symbiodiniaceae* with GF1 and cultured them for 3 months without antibiotics to stabilize the association with GF1 (Fig. 1a). Consequently, the resultant *Symbiodiniaceae* (Abx+GF1) showed a similar bacterial community (Fig. 1b and 3c) and stress tolerance to those of the control (Fig. 2a to e), suggesting that GF1 conferred stress tolerance to the cultured *Symbiodiniaceae*.

### GF1 produces zeaxanthin.

To investigate the possible mechanisms by which GF1 ameliorated oxidative damage (i.e., deterioration of F_v_/F_m_ and ROS production) in the cultured *Symbiodiniaceae*, we analyzed the metabolites produced in GF1, which accumulates orange pigments (Fig. 3a). Since bacteria in the genus *Muricauda* are characterized by the production of the natural xanthophyll pigment zeaxanthin (23, 24), which scavenges ROS and protects photosynthetic organisms from lipid peroxidation and photooxidative stress via nonphotochemical quenching (NPQ) (25, 26), we hypothesized that GF1 produced zeaxanthin, thus increasing the stress tolerance of the cultured *Symbiodiniaceae*.

**FIG 4:**
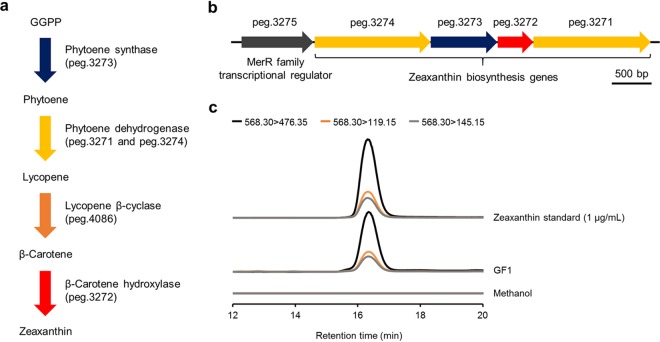
Zeaxanthin production by GF1. (a) Zeaxanthin biosynthesis pathway and corresponding genes of GF1. GGPP, geranylgeranyl diphosphate. (b) Zeaxanthin biosynthesis gene cluster. The annotation of each gene, which starts with “peg,” is listed in Table S4 in the supplemental material. (c) LC-MS/MS chromatograms of zeaxanthin in the methanol extract of GF1. Zeaxanthin was detected in positive ion mode by MRM from *m/z* 568.30 to 476.35, 119.15, and 145.15. A 1-μg/ml zeaxanthin standard is shown for retention time comparison. Zeaxanthin was not detected from the methanol used for extraction.

To test this hypothesis, we sequenced the genome of GF1 to determine whether this bacterium has the zeaxanthin biosynthesis pathway. A total of 63 contigs yielded a genome sequence (5,283,345 bp) with 85× coverage and an *N*_50_ value of 345,017. We confirmed the presence of all of the genes necessary for zeaxanthin biosynthesis in the GF1 genome (Fig. 4a; see also Table S4 in the supplemental material), encoding phytoene synthase (peg.3273), phytoene dehydrogenase (peg.3271 and peg.3274), lycopene β-cyclase (peg.4086), and β-carotene hydroxylase (peg.3272). All of the zeaxanthin biosynthesis genes, except for lycopene β-cyclase (peg.4086), were located downstream of a gene encoding a MerR family transcriptional regulator (peg.3275) with a vitamin B_12_-binding domain (Fig. 4b and Table S4), suggesting that carotenogenesis is regulated in response to photooxidative stress (27, 28). The biosynthesis pathways of other carotenoids were not confirmed in the GF1 genome, suggesting that zeaxanthin is most likely involved in stress mitigation. We further examined zeaxanthin production by GF1 at the metabolite level using liquid chromatography-tandem mass spectrometry (LC-MS/MS) in multiple reaction monitoring (MRM) mode (see Table S1). The yield of zeaxanthin was 8.03 ± 0.85 μg/g wet cell weight (Fig. 4c).

10.1128/mBio.01019-19.3 TABLE S1 MRM parameters for zeaxanthin analysis. Download Table S1, XLSX file, 0.1 MB. Copyright © 2020 Motone et al. 2020 Motone et al. This content is distributed under the terms of the Creative Commons Attribution 4.0 International license.

10.1128/mBio.01019-19.6 TABLE S4 Genome annotation of GF1 according to the RAST annotator. Download Table S4, XLSX file, 2.0 MB. Copyright © 2020 Motone et al. 2020 Motone et al. This content is distributed under the terms of the Creative Commons Attribution 4.0 International license.

### Zeaxanthin mitigates thermal and light stresses in cultured *Symbiodiniaceae*.

**FIG 5:**
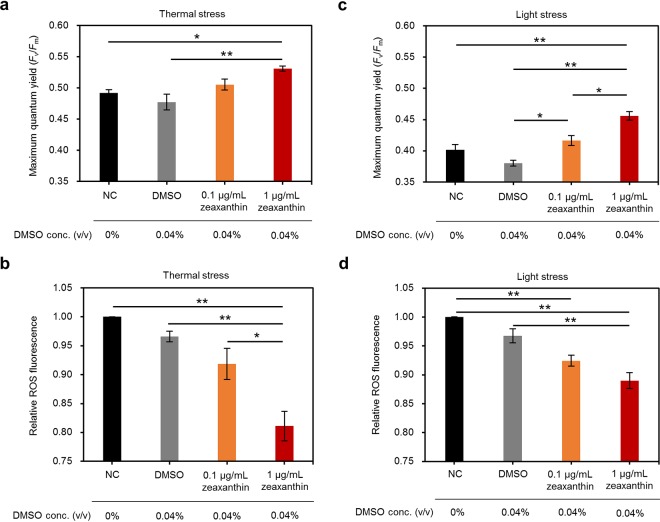
Effect of zeaxanthin supplementation on cultured *Symbiodiniaceae*. F_v_/F_m_ and relative ROS production in cultured *Symbiodiniaceae* supplemented with zeaxanthin under thermal stress (31.5°C, 50 μmol photons m^−2^ s^−1^) (a and b) and light stress (24°C, 200 μmol photons m^−2^ s^−1^) (c and d) conditions. (b, d) Data are provided as the relative fluorescence to NC. Error bars indicate SEMs from three biological replicates, and significant differences were determined by Tukey’s *post hoc* tests. *, *P* < 0.05; **, *P* < 0.01; NC, negative control (no supplementation); DMSO, dimethyl sulfoxide.

Next, we examined whether zeaxanthin supplementation to cultured *Symbiodiniaceae* can mitigate the oxidative damage induced by thermal and light stresses. Incubating the Abx algal culture with 1 μg/ml (1.76 μM) zeaxanthin resulted in significantly higher F_v_/F_m_ and lower ROS production under thermal stress (Fig. 5a and b). Under light stress, 0.1 and 1 μg/ml zeaxanthin supplementation significantly improved the F_v_/F_m_ and ameliorated ROS production (Fig. 5c and d). These results indicate that zeaxanthin is effective in mitigating thermal and light stresses and that cultured *Symbiodiniaceae* may benefit from the zeaxanthin supplied by GF1 under stressful conditions.

**FIG 6:**
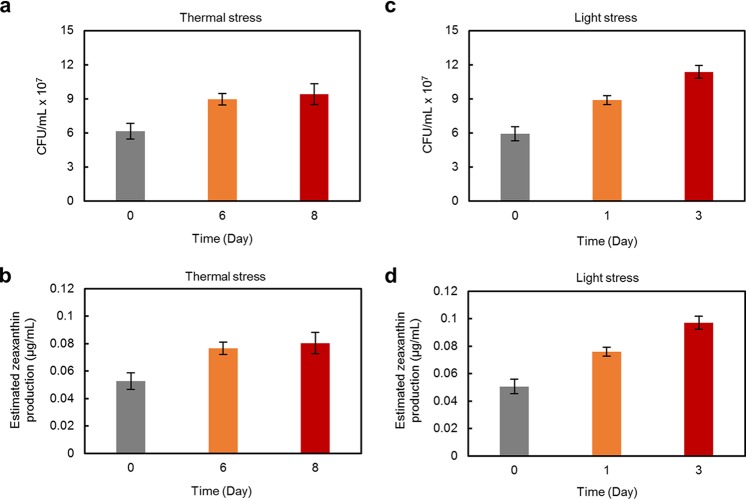
Estimation of zeaxanthin production. CFU/ml of GF1 in Abx+GF1 algal culture under thermal stress (31.5°C, 50 μmol photons m^−2^ s^−1^) (a) and light stress (24°C, 200 μmol photons m^−2^ s^−1^) (c) conditions. Estimated zeaxanthin productions by GF1 under thermal stress (b) and light stress (d) conditions. Zeaxanthin productions were calculated based on CFU of each condition. Error bars indicate SEMs from three biological replicates.

To investigate whether zeaxanthin dosages (0.1 and 1 μg/ml) are comparable to the zeaxanthin production by GF1, we performed colony-forming assays of GF1 in the Abx+GF1 algal culture and estimated the zeaxanthin production before and after stress challenges. The CFU of GF1 were 6.16 × 10^7^, 8.96 × 10^7^, and 9.40 × 10^7^ CFU/ml after 0-, 6-, and 8-day thermal stress exposure, respectively (Fig. 6a), and 5.92 × 10^7^, 8.88 × 10^7^, and 1.14 × 10^8^ CFU/ml after 0-, 1-, and 3-day light stress challenges, respectively (Fig. 6c). As 1 g wet cell weight of GF1 was equivalent to 9.39 × 10^9^ CFU and the yield of zeaxanthin was 8.03 μg/g wet cell weight (Fig. 4c), the zeaxanthin productions by GF1 in the algal culture were estimated to be 8.04 × 10^−2^ μg/ml after the 8-day thermal stress exposure (Fig. 6b) and 9.72 × 10^−2^ μg/ml after the 3-day light stress exposure (Fig. 6d). Given that 0.1 and 1 μg/ml of zeaxanthin supplementation are required to significantly reduce oxidative damage under light and thermal stress conditions, respectively (Fig. 5), GF1 was able to yield a comparable amount (9.72 × 10^−2^ μg/ml) of zeaxanthin required to mitigate light stress (0.1 μg/ml). Conversely, the estimated zeaxanthin production (8.04 × 10^−2^ μg/ml) was an order of magnitude less than the effective amount (1 μg/ml) under the thermal stress condition (Fig. 6b). This inconsistency suggests the presence of GF1-derived metabolites other than zeaxanthin that are also responsible for mitigating thermal stress and/or the potential importance of phycosphere interactions wherein the local concentration of zeaxanthin is significantly higher than the bulk concentration in the medium (29).

### *Flavobacteriaceae* bacteria are associated with cultured *Symbiodiniaceae* and corals.

**FIG 7:**
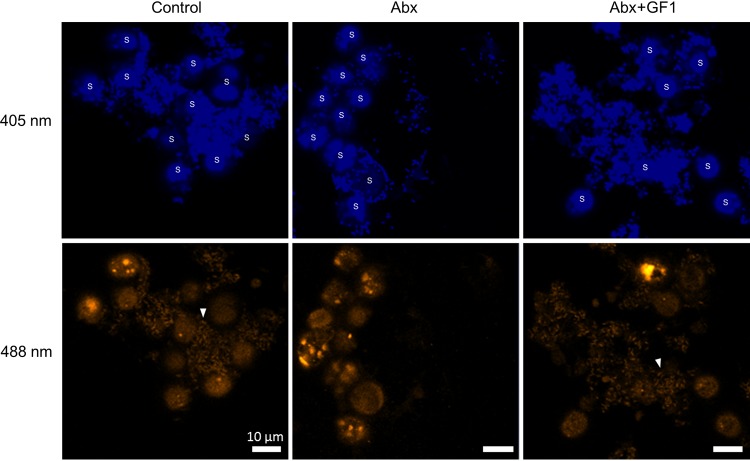
Fluorescence *in situ* hybridization (FISH) analysis of bacteria in algal cultures. DAPI and FAM fluorescence were detected by 405 and 488 nm excitation, respectively. *Symbiodiniaceae* cells were autofluorescent at 488 nm excitation. Arrowheads indicate the presence of rod-shaped bacteria hybridized with the CF319a probe labeled with FAM. S, *Symbiodiniaceae*; control, cultured *Symbiodiniaceae* without manipulation of bacterial community; Abx, cultured *Symbiodiniaceae* treated with antibiotics; Abx+GF1, cultured *Symbiodiniaceae* treated with antibiotics followed by inoculation of the bacterial strain GF1.

To determine whether there are close interactions between bacteria and algae, we performed fluorescence *in situ* hybridization (FISH) using a 6-carboxyfluorescein (FAM)-labeled oligonucleotide probe, CF319a (30), to specifically target the 16S rRNA sequence of *Flavobacteriaceae* bacteria (GF1). FAM signals were detected from bacteria in the vicinity of autofluorescent *Symbiodiniaceae* cells in the control and Abx+GF1 cultures but were absent in the Abx algal culture (Fig. 7). This result suggests that the signals were derived from *Flavobacteriaceae* bacteria (GF1) and that *Flavobacteriaceae* bacteria were intimately associated with *Symbiodiniaceae* rather than present only in the medium.

Moreover, we investigated whether *Flavobacteriaceae* bacteria are associated with corals. To this end, we isolated bacteria from coral tissue homogenates and sequenced the 16S rRNA genes of the bacteria. Consequently, 14.7% of all of the isolates that formed colonies on marine agar plates (19/129 colonies) showed a 16S rRNA gene sequence identical to that of GF1 (see Table S2), suggesting that zeaxanthin-producing bacteria are not only associated with cultured *Symbiodiniaceae* but also present in coral holobionts.

10.1128/mBio.01019-19.4 TABLE S2 Bacteria isolated from corals. Download Table S2, XLSX file, 0.1 MB. Copyright © 2020 Motone et al. 2020 Motone et al. This content is distributed under the terms of the Creative Commons Attribution 4.0 International license.

## DISCUSSION

Several studies have demonstrated that *Muricauda* bacteria are intimately associated with microalgae, including *Symbiodiniaceae* (19, 31). However, their functional roles have yet be investigated. This study revealed that GF1, a close relative to *M. lutaonensis*, contributes to enhancing the stress resistance of cultured *Symbiodiniaceae*. Interestingly, *Muricauda* is one of 12 bacterial genera ubiquitously associated with various reef-building corals across the globe, suggesting its functional importance not only in cultured *Symbiodiniaceae* but also in coral holobionts (18).

Zeaxanthin is a xanthophyll carotenoid present in photosynthetic organisms such as plants and algae (25, 32). In addition to its antioxidant activity, zeaxanthin can serve as a membrane stabilizer in chloroplasts to increase their thermal tolerance by decreasing membrane fluidity (33, 34). Zeaxanthin is also known to accumulate via the xanthophyll cycle in response to excess light exposure and to induce NPQ by dissipating excess excitation energy as heat (26). Since the xanthophyll cycle is composed of diadinoxanthin and diatoxanthin (which is functionally equivalent to zeaxanthin) in microalgae such as dinoflagellates and diatoms (35, 36), the zeaxanthin produced by GF1 could be converted into diatoxanthin in *Symbiodiniaceae* to induce NPQ, thereby protecting them from light stress. Although we hypothesized that GF1 contributes to the improved stress tolerance of cultured *Symbiodiniaceae* through zeaxanthin production, several peaks of metabolites other than zeaxanthin were also detected from the GF1 extract (see Fig. S1 in the supplemental material). These metabolites may also contribute to enhancing the stress tolerance of cultured *Symbiodiniaceae*. Further studies are warranted to scrutinize the dynamics of zeaxanthin and such metabolites in the phycosphere to determine the detailed mechanisms by which GF1 helps cultured *Symbiodiniaceae* cope with thermal and light stresses.

10.1128/mBio.01019-19.1 FIG S1 LC-UV-visible chromatograms of the methanol extract of GF1. Zeaxanthin and other metabolites were detected in GF1 at a wavelength of 450 nm. Samples were separated by an InertSustain AQ-C_18_ column at a column temperature of 35°C. The mobile phase was 90% (vol/vol) methanol at a flow rate of 0.4 ml/min. A 1-μg/ml zeaxanthin standard was shown for retention time comparison. Zeaxanthin was not detected from the methanol used for extraction. Download FIG S1, PDF file, 0.2 MB. Copyright © 2020 Motone et al. 2020 Motone et al. This content is distributed under the terms of the Creative Commons Attribution 4.0 International license.

We observed that *Alphaproteobacteria* (OTU1) were not eliminated from the *Symbiodiniaceae* culture by antibiotics, which is consistent with a previous report (20). Since the relative abundance of *Alphaproteobacteria* was higher in the Abx group than in the control and Abx+GF1 groups (Fig. 1b), it is difficult to completely exclude the possibility that the *Alphaproteobacteria* are opportunistic and increase algal oxidative stress upon environmental stress, e.g., by producing virulence factors. To our knowledge, Vibrio shilonii (37) and Vibrio coralliilyticus (38) are the only pathogens that have been shown to cause photoinhibition in *Symbiodiniaceae*. In contrast, *Alphaproteobacteria* are assumed to have beneficial functions such as growth promotion, nutrient cycling, and production of bioactive compounds (e.g., vitamin B_12_) that are most likely essential for the survival of cultured *Symbiodiniaceae* (39–42). Indeed, the 16S rRNA gene sequence of an alphaproteobacterium isolated from an algal culture in the present study showed the highest similarity (99.3%) to that of a *Symbiodiniaceae*-associated bacterium, alpha proteobacterium Mf 1.05b.01 (20), which possesses a predicted vitamin B_12_ biosynthesis pathway (see Fig. S2). Further microbiome manipulations such as phage treatment (43, 44) for the elimination of *Alphaproteobacteria* are required to ascertain whether *Alphaproteobacteria* (OTU1) are beneficial to cultured *Symbiodiniaceae*.

10.1128/mBio.01019-19.2 FIG S2 Putative vitamin B_12_ biosynthesis genes in the genome of alpha proteobacterium Mf 1.05b.01. The annotation of each gene, which starts with “peg,” is listed in Table S5. BtuB is a TonB-dependent transporter located in the outer membrane. BtuF is a periplasmic corrinoid-binding protein. BtuC and BtuD are a membrane permease and an ATPase, respectively. FMN, flavin mononucleotide. Download FIG S2, PDF file, 0.1 MB. Copyright © 2020 Motone et al. 2020 Motone et al. This content is distributed under the terms of the Creative Commons Attribution 4.0 International license.

To isolate *Symbiodiniaceae* from corals, we used antibiotics to suppress the growth of bacteria; otherwise, the algal cells would not have been able to grow (21). Therefore, the bacterial community of the control algae was perturbed, and some bacteria with important functions for *Symbiodiniaceae* may have been eliminated at the start of the stress experiments. Techniques such as droplet microfluidics may enable the isolation and cultivation of *Symbiodiniaceae* without antibiotic usage to facilitate the analysis of interactions between *Symbiodiniaceae* and an intact bacterial community. Although the antibiotic treatment definitely brought about an artificial effect on the bacterial community of the control algae, which showed low bacterial diversity, this enabled us to disentangle the complex interactions between *Symbiodiniaceae* and bacteria. Since, compared with that of corals, the bacterial communities of algal cultures are simple and amenable to manipulation through antibiotic treatment and bacterial inoculation, as shown in the present and previous studies (17, 40, 45), cultured *Symbiodiniaceae* can be used as a platform for initial screenings and functional assessments of bacteria that are beneficial or harmful to *Symbiodiniaceae* and the coral holobiont.

Future studies should focus on determining the functions and localizations of zeaxanthin-producing bacteria in the coral holobiont. For example, whether zeaxanthin-producing bacteria are present within the coral tissues would be a major determinant of their ROS-scavenging capabilities, given that ROS production on endosymbiotic algae would first affect the gastrodermal cells of the coral host. In conclusion, this study illuminates the beneficial effects of bacteria on cultured *Symbiodiniaceae* and contributes to the understanding and enhancing of the stress resistance of the coral holobiont, which is facing unprecedented environmental stress.

## MATERIALS AND METHODS

### Coral culture and *Symbiodiniaceae* isolation.

The *G. fascicularis* colonies were purchased from Shimizu Laboratory Supplies (Kyoto, Japan) and kept at 24°C in an aquarium (Shimizu Laboratory Supplies) containing ∼200 liters of artificial seawater prepared with Coral Pro Salt (Red Sea, Houston, TX, USA). The aquarium was equipped with a light-emitting diode (LED) lamp (KR90DR; Blue Harbor, Osaka, Japan), and 10% of the seawater was replaced every 3 to 4 days.

To isolate *Symbiodiniaceae*, a *G. fascicularis* colony was fragmented to obtain a single polyp and centrifuged at 100 × *g* for 10 s in a 1.5-ml tube to remove surface-associated seawater. The tissue of the polyp was harvested by further centrifugation at 8,000 × *g* for 2 min and suspended in 1 ml of f/2 liquid medium. Then, the tissue suspension was serially diluted and incubated on f/2 agar plates supplemented with antibiotics (50 μg/ml of kanamycin, 100 μg/ml of ampicillin, and 50 μg/ml of streptomycin) (21) at 24°C and a light intensity of 50 μmol photons m^−2^ s^−1^ on a 14-h:10-h light/dark cycle for 30 days. The grown colonies were subsequently incubated in f/2 liquid medium without antibiotics at the same conditions described above and subcultured every 30 to 50 days until the start of the experimental trials. A photon sensor (DEFI2-L; JFE Advantech, Nishinomiya, Japan) was used to measure light intensity. All of the *Symbiodiniaceae* isolates were identified as *Durusdinium* (formerly *Symbiodinium* clade D) (46) based on the internal transcribed spacer 2 (ITS2) region sequences (47).

### Manipulation of the bacterial community of *Symbiodiniaceae*.

The bacterial community of *Symbiodiniaceae* was perturbed by incubating an algal culture for 2 months in f/2 liquid medium supplemented with the antibiotic cocktail described above. To isolate a bacterium from the cultured *Symbiodiniaceae*, the algal suspension was serially diluted and spread on marine agar plates (BD Biosciences, Franklin Lakes, NJ, USA). After incubation at 25°C for 5 days, a colony showing orange pigmentation was isolated, purified, and named GF1. The antibiotic-treated *Symbiodiniaceae* (∼10^5^ cells) were inoculated with ∼10^6^ CFU of GF1 and cultured in the absence of antibiotics for at least 3 months to stabilize the association with *Symbiodiniaceae* before being used in subsequent experiments.

### 16S rRNA amplicon sequencing and data analysis.

*Symbiodiniaceae* cells were harvested via centrifugation at 8,000 × *g* for 5 min and immediately stored at −80°C until processing. The total DNAs were extracted using the PowerSoil DNA Isolation kit (Qiagen, Hilden, Germany) according to the manufacturer’s instructions. The variable region V1/V2 of the 16S rRNA gene was amplified using the primers 8F-overhang (5ʹ-TCGTCGGCAGCGTCAGATGTGTATAAGAGACAGAGAGTTTGATCMTGGCTCAG-3ʹ) and 338R-overhang (5ʹ-GTCTCGTGGGCTCGGAGATGTGTATAAGAGACAGTGCTGCCTCCCGTAGGAGT-3ʹ). PCR amplifications were performed in a final volume of 25 μl, containing 12.5 μl of KAPA HiFi HotStart ReadyMix, 0.75 μl of each primer (10 μM), and 3 ng of template DNA. The PCR cycling conditions were as follows: initial denaturing at 95°C for 4 min and then 23 cycles of 98°C for 20 s, 55°C for 15 s, and 72°C for 15 s, followed by a final extension at 72°C for 4 min. The amplicons were subsequently indexed using the Nextera XT Index kit (Illumina, San Diego, CA, USA) and sequenced on the Illumina Miseq platform using 2 × 250-bp paired-end v2 chemistry.

For the taxonomic analysis, the forward reads were cropped to 240 bp using Trimmomatic (48) and subsequently processed with the QIIME pipeline (49). Briefly, low-quality reads with a Qscore of less than 20 and chimeric sequences were discarded. The surviving sequences (ranging from 27,314 to 100,687 reads per sample) were then clustered into operational taxonomic units (OTUs) and classified using the Greengenes database via open-reference OTU picking with the default parameters (see Table S3 in the supplemental material). The bacterial community composition and weighted UniFrac distances were calculated after removing the OTUs assigned to chloroplasts.

10.1128/mBio.01019-19.5 TABLE S3 OTU sequence counts, taxonomic classification, and 16S rRNA representative sequence. Download Table S3, XLSX file, 0.1 MB. Copyright © 2020 Motone et al. 2020 Motone et al. This content is distributed under the terms of the Creative Commons Attribution 4.0 International license.

### Phylogenetic analysis of GF1.

Genomic DNA was isolated from GF1 grown on marine agar plates using the DNeasy Blood & Tissue kit (Qiagen). The 16S rRNA gene of GF1 was PCR-amplified using KOD FX Neo (TOYOBO, Osaka, Japan) and the primers 8F (5ʹ-AGAGTTTGATCMTGGCTCAG-3ʹ) and 1492R (5ʹ-GGTTACCTTGTTACGACTT-3ʹ) according to the manufacturer’s instructions. The PCR cycling conditions were as follows: initial denaturing at 94°C for 2 min and then 30 cycles of 98°C for 10 s, 55°C for 30 s, and 68°C for 80 s, followed by a final extension at 68°C for 3 min. The 16S rRNA gene of GF1 was sequenced (Eurofins Genomics, Ebersberg, Germany). Other bacterial sequences were obtained through EzBioCloud (50). The phylogenetic tree was constructed based on the 16S rRNA sequences via the neighbor-joining method (bootstrap of 1,000 replicates) using Clustal X (http://www.clustal.org/).

### Stress challenges and physiological assessment.

*Symbiodiniaceae* cells were acclimatized in 5 ml f/2 medium in a 14-ml round-bottom tube (352059; Corning, Corning, NY, USA) under the same culture conditions described above for at least 5 days before stress exposure. For the heat stress challenge, *Symbiodiniaceae* cells at an initial cell density of 10^6^ cells/ml were exposed to thermal (31.5°C) or nonthermal (24°C) stress at a light intensity of 50 μmol photons m^−2^ s^−1^. For the light stress experiment, the *Symbiodiniaceae* cells were incubated at 24°C under light (200 μmol photons m^−2^ s^−1^) or nonlight (50 μmol photons m^−2^ s^−1^) stress. The F_v_/F_m_ was measured from the bottom of the tubes using a pulse-amplitude-modulated fluorometer (Junior-PAM; Walz, Effeltrich, Germany) after the algal cultures had been dark adapted for 30 min. To quantify the ROS production in the algal cultures, 195 μl of algal suspension was incubated with 5 μl of 2ʹ,7ʹ-dichlorofluorescin diacetate (Sigma-Aldrich, St. Louis, MO, USA; 2 mM in dimethyl sulfoxide) in a 96-well plate for ∼30 min in darkness, followed by measurement of the fluorescence intensity (excitation at 485 nm and emission at 527 nm) using a microplate fluorometer (Fluoroskan Ascent FL; Thermo Fisher Scientific, Waltham, MA, USA). The data were represented as relative fluorescence normalized by algal cell numbers after subtracting the fluorescence intensity in f/2 medium without algal cells.

### Genome sequencing and analysis of GF1.

A sequencing library was prepared from the genomic DNA of GF1 using the Nextera DNA Library Preparation kit (Illumina) according to the manufacturer’s protocol and sequenced on the Illumina Miseq platform using 2 × 150-bp paired-end v2 chemistry. The raw sequencing reads were filtered using fastp (version 0.20.0) (51) with the default parameters and subsequently *de novo* assembled using SPAdes (version 3.13.1) (52) with the parameters “–careful” and “–cov-cutoff auto.” The assembled genome was annotated using the RAST server (version 2.0) (53). All of the coding sequences and annotation information are listed in Table S4. The zeaxanthin biosynthesis pathway was confirmed based on the RAST and KAAS annotators (54).

### Metabolite analysis.

Pigments were extracted from ∼10 mg wet cell weight of GF1 grown on marine agar plates with methanol using a Bioruptor UCD-250 sonicator (Cosmo Bio, Tokyo, Japan) for 10 s. The LC-MS/MS analysis was performed using LC (Nexera System; Shimadzu, Kyoto, Japan) triple quadrupole mass spectrometry (LCMS-8060; Shimadzu). The samples were separated by an InertSustain AQ-C_18_ column (150 mm by 2.1 mm inside diameter [i.d.] and 1.9-μm particle size; GL Sciences, Osaka, Japan) at a column temperature of 35°C. The mobile phase comprised 90% (vol/vol) methanol containing 0.1% (vol/vol) formic acid at a flow rate of 0.4 ml/min. Electrospray ionization was performed at 4 kV, with the positive mode at 250°C in the desolvation line and 300°C in the interface. Nebulizing and drying gases were set at flow rates of 2 and 10 liters/min, respectively. Data acquisition was performed based on the MRM mode (Table S1), and the zeaxanthin abundance was determined on the basis of the peak area of the transition from *m/z* 568.30 to 476.35. A zeaxanthin standard was purchased from Sigma-Aldrich.

### Zeaxanthin addition experiments.

A stock solution of zeaxanthin (Cayman Chemical Company, Ann Arbor, MI, USA) was prepared in dimethyl sulfoxide. After acclimation under nonstressful conditions (24°C, 50 μmol photons m^−2^ s^−1^) for 5 days, the Abx algal culture was exposed to either thermal stress (31.5°C, 50 μmol photons m^−2^ s^−1^) for 6 days or light stress (24°C, 200 μmol photons m^−2^ s^−1^) for 1 day to induce oxidative damage. Subsequently, the Abx algal culture was supplemented with zeaxanthin to a final concentration of 0.1 or 1 μg/ml and incubated for 2 days under the same stressful conditions followed by F_v_/F_m_ and ROS production measurements as described above.

### Colony-forming assays.

Colony-forming assays were performed to estimate the zeaxanthin production by GF1 in Abx+GF1 algal cultures. Algal cultures before and after stress exposure were serially diluted in f/2 medium and spread on marine agar plates to form 30 to 100 bacterial colonies. The numbers of colonies were counted after incubation at 25°C for 6 days.

### FISH analysis.

All procedures were performed according to the method described elsewhere (55). In brief, algal samples were fixed in 4% paraformaldehyde (Wako, Osaka Japan), spotted on an aminosilane-coated glass slide (Matsunami Glass, Osaka, Japan), and air-dried, followed by successive dehydration in 50%, 80%, and 100% ethanol. The samples were hybridized with a CF319a probe (5ʹ-TGGTCCGTGTCTCAGTAC-3ʹ) labeled with the fluorophore FAM (Eurofins Genomics) in a hybridization buffer (35% formamide, 0.9 M NaCl, 20 mM Tris-HCl, and 0.01% SDS) at 46°C for 2 h. After being washed with a washing buffer (0.08 M NaCl, 20 mM Tris-HCl, 5 mM EDTA, and 0.01% SDS), the samples were counterstained with DAPI (4′,6-diamidino-2-phenylindole; Nacalai Tesque, Kyoto, Japan) in Vectashield mounting medium (Vector Laboratories, CA, USA) and observed under a confocal microscope (LSM 700; Zeiss, Oberkochen, Germany).

### Isolation of bacteria from corals.

The *G. fascicularis* colonies were fragmented to obtain single polyps and centrifuged at 100 × *g* for 10 s in a 1.5-ml tube to remove surface-associated seawater. The tissues of the polyps were harvested via further centrifugation at 8,000 × *g* for 2 min and homogenized in marine broth (BD Biosciences). The coral tissue suspensions were serially diluted and spread on marine agar plates to form 30 to 100 bacterial colonies. Among the colonies formed during the 10-day incubation at 25°C, those showing orange pigmentation similar to that of GF1 were directly subjected to PCR amplifications as described above. The amplified 16S rRNA genes of the bacteria were then sequenced (Eurofins Genomics) and compared with that of GF1.

### Genome analysis of a closely related alphaproteobacterium.

To isolate an alphaproteobacterium from the cultured *Symbiodiniaceae*, the Abx algal culture was spread on a marine agar plate. After incubation at 25°C for 10 days, a white colony was isolated and incubated at 98°C for 5 min in ultrapure water to extract DNA. After centrifugation at 14,000 × *g* for 1 min, the supernatant was used as a DNA template for PCR using 8F and 1492R primers. The amplified 16S rRNA gene was sequenced (Eurofins Genomics). The sequence was identical to the representative sequence of OTU1 (Table S3).

To infer the functional roles of the alphaproteobacterium, the genome sequence of a closely related strain, alpha proteobacterium Mf 1.05b.01, was downloaded from NCBI (NZ_BAOK00000000.1) (20) and annotated using the RAST server (53). All of the coding sequences and annotation information are listed in Table S5. The presence of the vitamin B_12_ biosynthesis pathway was confirmed according to the RAST and KAAS annotators (54).

10.1128/mBio.01019-19.7 TABLE S5 Genome annotation of alpha proteobacterium Mf 1.05b.01 according to the RAST annotator. Download Table S5, XLSX file, 1.5 MB. Copyright © 2020 Motone et al. 2020 Motone et al. This content is distributed under the terms of the Creative Commons Attribution 4.0 International license.

### Data availability.

The 16S rRNA amplicon sequencing data were deposited in the NCBI Sequence Read Archive under accession number PRJNA514199. The 16S rRNA gene of GF1 was deposited in GenBank under accession number MK391175. The GF1 genome sequencing data were deposited in GenBank under accession number PRJNA588666. The 16S rRNA gene of the alphaproteobacterium isolated in the present study was deposited in GenBank under accession number MN658478.
