# Supplementary material for: A Zeaxanthin-Producing Bacterium Isolated from the Algal Phycosphere Protects Coral Endosymbionts from Environmental Stress
Source: mBio. 2020 Jan 21;11(1):e01019-19. doi: 10.1128/mBio.01019-19 (PMC6974559; doi:10.1128/mBio.01019-19)
Supplement: FIG S1 [file mBio.01019-19-sf001.pdf]

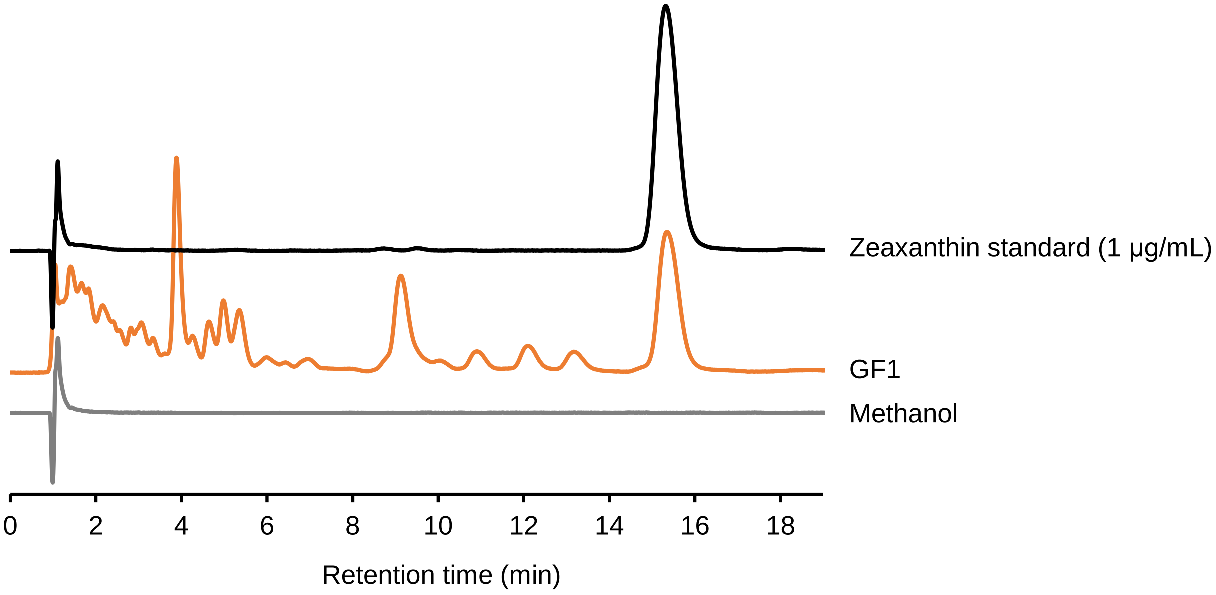

Fig. S1 LC–UV/Vis chromatograms of the methanol extract of GF1. Zeaxanthin and other metabolites were detected in GF1 at a wavelength of 450 nm. Samples were separated by an InertSustain AQ-C18 column at a column temperature of 35°C. The mobile phase was 90% (v/v) methanol at a flow rate of 0.4 mL/min. A 1 μg/mL zeaxanthin standard was shown for retention time comparison. Zeaxanthin was not detected from the methanol used for extraction.
