# Supplementary material for: A Zeaxanthin-Producing Bacterium Isolated from the Algal Phycosphere Protects Coral Endosymbionts from Environmental Stress
Source: mBio. 2020 Jan 21;11(1):e01019-19. doi: 10.1128/mBio.01019-19 (PMC6974559; doi:10.1128/mBio.01019-19)
Supplement: FIG S2 [file mBio.01019-19-sf002.pdf]

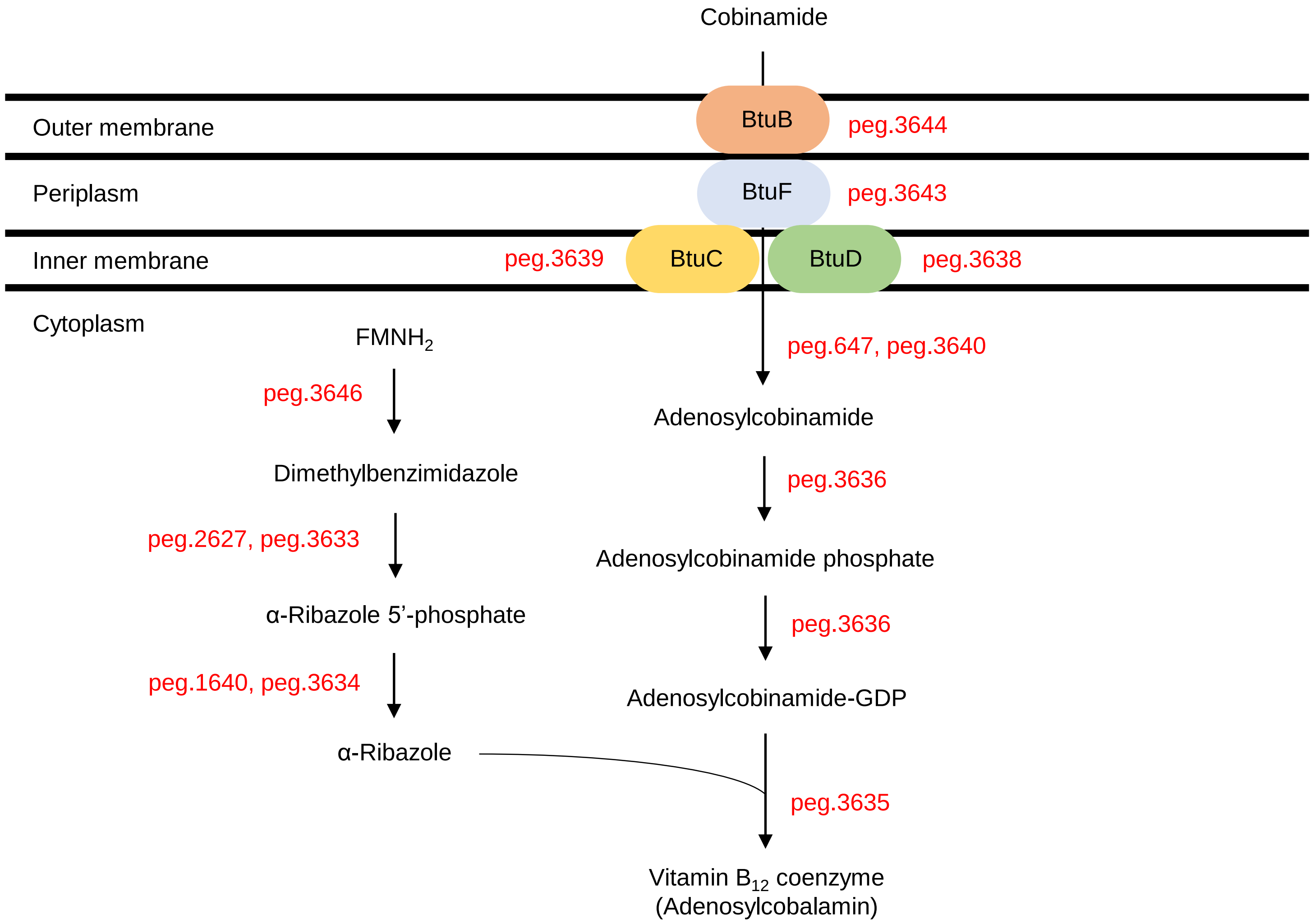

Fig. S2 Putative vitamin B$_{12}$ biosynthesis genes in the genome of alpha proteobacterium Mf 1.05b.01. The annotation of each gene, which is started with "peg," is listed in Table S5. BtuB is a TonB-dependent transporter located in the outer membrane. BtuF is a periplasmic corrinoid-binding protein. BtuC and BtuD are a membrane permease and an ATPase, respectively. FMN, Flavin mononucleotide; GDP, Guanosine diphosphate.
